# Poly[[μ-ethane-1,2-diyl bis­(pyridine-3-carboxyl­ate)](μ-tetra­fluorido­borato)silver(I)]

**DOI:** 10.1107/S1600536812018399

**Published:** 2012-04-28

**Authors:** Javier Vallejos, Iván Brito, Alejandro Cárdenas, Michael Bolte

**Affiliations:** a Departamento de Química, Facultad de Ciencias Básicas, Universidad de Antofagasta, Casilla 170, Antofagasta, Chile; b Departamento de Física, Facultad de Ciencias Básicas, Universidad de Antofagasta, Casilla 170, Antofagasta, Chile; c Institut für Anorganische Chemie der Goethe-Universität Frankfurt, Max-von-Laue-Strasse 7, D-60438 Frankfurt am Main, Germany

## Abstract

In the title compound, [Ag(BF_4_)(C_14_H_12_N_2_O_4_)]_*n*_, the coordination of the Ag^+^ ion is trigonal–bipyramidal with the N atoms of two ethane-1,2-diyl bis­(pyridine-3-carboxyl­ate) ligands in the apical positions and three F atoms belonging to different tetra­fluorido­borate anions in the equatorial plane. The material consists of infinite chains of [Ag(C_14_H_12_N_2_O_4_)] units running along [001], held together by BF_4_^−^ bridging anions.

## Related literature

For the crystal structure of the ethane-1,2-diyl bis­(pyridine-3-carboxyl­ate) ligand, see: Brito *et al.* (2010 ▶). For background to coordination chemistry, see: Blake *et al.* (1999 ▶); Brito *et al.* (2011 ▶). 
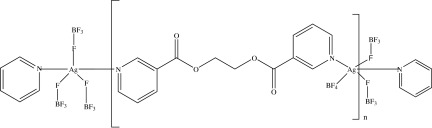


## Experimental

### 

#### Crystal data

[Ag(BF_4_)(C_14_H_12_N_2_O_4_)]
*M* *_r_* = 466.94
Orthorhombic, 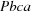

*a* = 15.2667 (11) Å
*b* = 6.7170 (4) Å
*c* = 30.8598 (16) Å
*V* = 3164.6 (3) Å^3^
*Z* = 8
Mo *K*α radiation
μ = 1.34 mm^−1^
*T* = 173 K
0.16 × 0.04 × 0.04 mm

#### Data collection

Stoe IPDS-II two-circle diffractometer
Absorption correction: multi-scan (*MULABS*; Spek, 2009 ▶; Blessing, 1995 ▶) *T*_min_ = 0.814, *T*_max_ = 0.948
27909 measured reflections
2772 independent reflections
1605 reflections with *I* > 2σ(*I*)
*R*_int_ = 0.115

#### Refinement

*R*[*F* ^2^ > 2σ(*F* ^2^)] = 0.058
*wR*(*F* ^2^) = 0.144
*S* = 0.93
2772 reflections
235 parameters
H-atom parameters constrained
Δρ_max_ = 1.75 e Å^−3^
Δρ_min_ = −1.14 e Å^−3^

### 

Data collection: *X-AREA* (Stoe & Cie, 2001 ▶); cell refinement: *X-AREA*; data reduction: *X-AREA*; program(s) used to solve structure: *SHELXS97* (Sheldrick, 2008 ▶); program(s) used to refine structure: *SHELXL97* (Sheldrick, 2008 ▶); molecular graphics: *XP* in *SHELXTL* (Sheldrick, 2008 ▶); software used to prepare material for publication: *SHELXL97*.

## Supplementary Material

Crystal structure: contains datablock(s) I, global. DOI: 10.1107/S1600536812018399/ff2064sup1.cif

Structure factors: contains datablock(s) I. DOI: 10.1107/S1600536812018399/ff2064Isup2.hkl

Additional supplementary materials:  crystallographic information; 3D view; checkCIF report

## Figures and Tables

**Table 1:** Selected bond lengths (Å)

Ag1—N11	2.145 (6)
Ag1—N23^i^	2.155 (6)
Ag1—F1^ii^	3.168 (9)
Ag1—F2	2.832 (8)
Ag1—F2^iii^	2.972 (7)

Symmetry codes: (i) 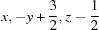; (ii) 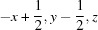; (iii) 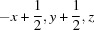.

**Fig. 1.**
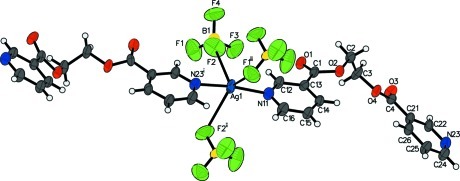
Part of the polymeric structure and the atom-numbering scheme of the title compound. Displacement ellipsoids are shown at the 30% probability level. [Symmetry codes (i) x, 3/2 - y, -1/2 + z; (ii) 1/2 - x, 1/2 + y, z; (iii) 1/2 - x, -1/2 + y, z].

**Fig. 2.**
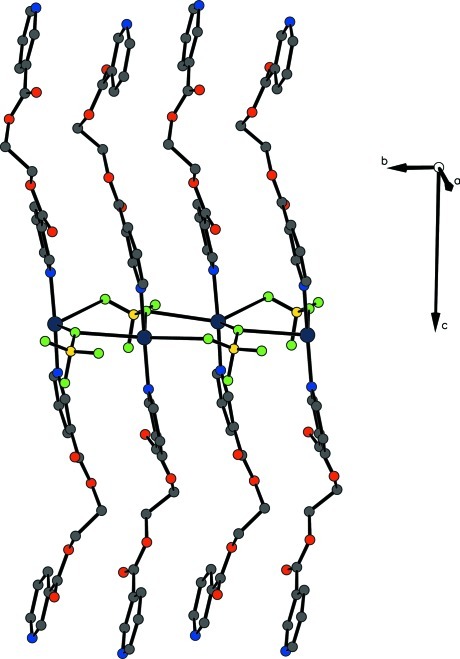
Infinite chains of [Ag(C14H12N2O4)] moieties running along [001] held together by BF4- bridging anions. The H-toms have been omitted for clarity.

## supplementary crystallographic information

### Comment

The design of polymeric organic-inorganic materials with novel topologies and structural motifs is of current interest in the field of coordination chemistry (Blake *et al.*, 1999). This paper forms part of our continuing study of the synthesis, structural characterization and physical properties of coordination polymers (Brito *et al.*, 2011). We report here the crystal structure of the title compound (Fig.1). It crystallizes as colorless needles, which were found to be stable to air and light. In the title compound, the coordination of the Ag^I^ atom is a trigonal bipyramid with the N atoms of two 1,2-diyl-bis(pyridine-3-carboxylate)ethane ligands in the apical positions and three F atoms belonging to different tetrafluoroborate anions in the equatorial plane. The Ag–N distances are 2.145 (6) and 2.155 (6) Å and the Ag–F distances are 2.832 (8) Å, 2.972 (7) Å and 3.168 (9) Å. The N–Ag–N angle [168.8 (3)°] is not far from being linear and the Ag—N vectors are almost perpendicular to the Ag—F vectors [76.7 (3)° to 103.1 (3)°]. The F–Ag–F angles are 106.4 (2)°, 120.7 (2)° and 127.4 (2)°. The molecular dimensions of the 1,2-bis(3-pyridyl)ethane ligand are within normal ranges and the ethylene moiety retains the *gauche* conformation (Brito *et al.*, 2010). The material consists of infinite chains of [Ag(C_14_H_12_N_2_O_4_)] moieties running along [001] held together by BF_4_^-^ bridging anions (Fig. 2).

### Experimental

A solution of AgBF_4_ (19.4 mg, 0.1 mmol) in water was slowly added to a solution of the ligand (27.2 mg, 0.1 mmol) in THF (4 ml). Colorless single crystals suitable for X-ray were obtained after a few days (82%).The FT—IR (KBr, cm^-1^): 1724 s, 1609, 1434 m, 1280 s, 742 m.

### Refinement

All H-atoms were positioned geometrically with C—H in the range of 0.95 or 0.99 Å and refined using a riding model with *U*_iso_(H)= 1.2 *U*_eq_(C).

### Crystal data

**Table:**

[Ag(BF_4_)(C_14_H_12_N_2_O_4_)]	*F*(000) = 1840
*M**_r_* = 466.94	*D*_x_ = 1.960 Mg m^−^^3^
Orthorhombic, *P**b**c**a*	Mo *K*α radiation, λ = 0.71073 Å
Hall symbol: -P 2ac 2ab	Cell parameters from 9495 reflections
*a* = 15.2667 (11) Å	θ = 2.7–25.9°
*b* = 6.7170 (4) Å	µ = 1.34 mm^−^^1^
*c* = 30.8598 (16) Å	*T* = 173 K
*V* = 3164.6 (3) Å^3^	Needle, colourless
*Z* = 8	0.16 × 0.04 × 0.04 mm

### Data collection

**Table:**

Stoe IPDS-II two-circle diffractometer	2772 independent reflections
Radiation source: fine-focus sealed tube	1605 reflections with *I* > 2σ(*I*)
Graphite monochromator	*R*_int_ = 0.115
ω scans	θ_max_ = 25.0°, θ_min_ = 2.6°
Absorption correction: multi-scan (*MULABS*; Spek, 2009; Blessing, 1995)	*h* = −18→18
*T*_min_ = 0.814, *T*_max_ = 0.948	*k* = −7→7
27909 measured reflections	*l* = −36→36

### Refinement

**Table:**

Refinement on *F*^2^	Primary atom site location: structure-invariant direct methods
Least-squares matrix: full	Secondary atom site location: difference Fourier map
*R*[*F*^2^ > 2σ(*F*^2^)] = 0.058	Hydrogen site location: inferred from neighbouring sites
*wR*(*F*^2^) = 0.144	H-atom parameters constrained
*S* = 0.93	*w* = 1/[σ^2^(*F*_o_^2^) + (0.0668*P*)^2^] where *P* = (*F*_o_^2^ + 2*F*_c_^2^)/3
2772 reflections	(Δ/σ)_max_ < 0.001
235 parameters	Δρ_max_ = 1.75 e Å^−^^3^
0 restraints	Δρ_min_ = −1.14 e Å^−^^3^

### Special details

**Table:**

Geometry. All e.s.d.'s (except the e.s.d. in the dihedral angle between two l.s. planes) are estimated using the full covariance matrix. The cell e.s.d.'s are taken into account individually in the estimation of e.s.d.'s in distances, angles and torsion angles; correlations between e.s.d.'s in cell parameters are only used when they are defined by crystal symmetry. An approximate (isotropic) treatment of cell e.s.d.'s is used for estimating e.s.d.'s involving l.s. planes.
Refinement. Refinement of *F*^2^ against ALL reflections. The weighted *R*-factor *wR* and goodness of fit *S* are based on *F*^2^, conventional *R*-factors *R* are based on *F*, with *F* set to zero for negative *F*^2^. The threshold expression of *F*^2^ > σ(*F*^2^) is used only for calculating *R*-factors(gt) *etc*. and is not relevant to the choice of reflections for refinement. *R*-factors based on *F*^2^ are statistically about twice as large as those based on *F*, and *R*- factors based on ALL data will be even larger.

### Fractional atomic coordinates and isotropic or equivalent isotropic displacement parameters (Å^2^)

**Table:**

	*x*	*y*	*z*	*U*_iso_*/*U*_eq_	
Ag1	0.32630 (5)	0.68683 (11)	0.122155 (18)	0.0436 (2)	
O1	0.1384 (4)	0.6081 (9)	0.27974 (18)	0.0388 (14)	
O2	0.2325 (3)	0.4748 (8)	0.32836 (16)	0.0318 (13)	
O3	0.1274 (4)	0.7200 (9)	0.46953 (18)	0.0426 (15)	
O4	0.2171 (3)	0.6095 (9)	0.41696 (16)	0.0350 (13)	
C1	0.2118 (6)	0.5608 (13)	0.2904 (2)	0.0318 (19)	
C2	0.1594 (5)	0.4311 (13)	0.3564 (2)	0.0347 (19)	
H2A	0.1715	0.3060	0.3723	0.042*	
H2B	0.1065	0.4096	0.3385	0.042*	
C3	0.1415 (5)	0.5929 (15)	0.3883 (2)	0.038 (2)	
H3A	0.1317	0.7205	0.3730	0.046*	
H3B	0.0884	0.5611	0.4054	0.046*	
C4	0.1992 (5)	0.6804 (12)	0.4566 (2)	0.0305 (17)	
N11	0.3421 (4)	0.6597 (11)	0.1910 (2)	0.0385 (17)	
C12	0.2759 (6)	0.6318 (12)	0.2184 (2)	0.035 (2)	
H12	0.2174	0.6408	0.2080	0.041*	
C13	0.2907 (5)	0.5899 (12)	0.2618 (2)	0.0287 (17)	
C14	0.3766 (5)	0.5786 (12)	0.2780 (3)	0.0310 (18)	
H14	0.3881	0.5471	0.3074	0.037*	
C15	0.4436 (5)	0.6155 (12)	0.2490 (3)	0.036 (2)	
H15	0.5027	0.6141	0.2586	0.044*	
C16	0.4251 (6)	0.6540 (14)	0.2065 (3)	0.042 (2)	
H16	0.4723	0.6777	0.1871	0.050*	
C21	0.2811 (5)	0.7043 (13)	0.4833 (2)	0.0295 (17)	
C22	0.2702 (6)	0.7513 (12)	0.5268 (2)	0.032 (2)	
H22	0.2126	0.7616	0.5382	0.039*	
N23	0.3379 (5)	0.7822 (10)	0.5529 (2)	0.0384 (17)	
C24	0.4197 (6)	0.7680 (13)	0.5363 (3)	0.041 (2)	
H24	0.4682	0.7907	0.5549	0.049*	
C25	0.4354 (6)	0.7217 (14)	0.4934 (3)	0.040 (2)	
H25	0.4936	0.7149	0.4827	0.048*	
C26	0.3651 (5)	0.6855 (13)	0.4662 (2)	0.0332 (17)	
H26	0.3739	0.6490	0.4367	0.040*	
B1	0.0651 (6)	0.6709 (18)	0.1252 (3)	0.040 (2)	
F1	0.0369 (5)	0.8388 (12)	0.1072 (3)	0.107 (3)	
F2	0.1447 (5)	0.6209 (11)	0.1101 (3)	0.098 (3)	
F3	0.0720 (6)	0.7014 (14)	0.1693 (2)	0.110 (3)	
F4	0.0034 (5)	0.5200 (11)	0.1204 (3)	0.097 (2)	

### Atomic displacement parameters (Å^2^)

**Table:**

	*U*^11^	*U*^22^	*U*^33^	*U*^12^	*U*^13^	*U*^23^
Ag1	0.0429 (4)	0.0680 (5)	0.0198 (3)	−0.0014 (4)	−0.0011 (3)	0.0042 (3)
O1	0.021 (3)	0.065 (4)	0.030 (3)	−0.002 (3)	−0.003 (2)	0.007 (3)
O2	0.030 (3)	0.046 (4)	0.019 (3)	−0.002 (2)	0.003 (2)	0.003 (2)
O3	0.026 (3)	0.068 (4)	0.033 (3)	0.005 (3)	0.003 (3)	−0.012 (3)
O4	0.029 (3)	0.059 (4)	0.018 (3)	−0.002 (2)	−0.002 (2)	−0.002 (2)
C1	0.035 (5)	0.040 (5)	0.020 (4)	−0.003 (4)	−0.003 (3)	−0.001 (4)
C2	0.032 (5)	0.054 (5)	0.017 (4)	−0.009 (4)	0.006 (3)	−0.001 (4)
C3	0.024 (4)	0.067 (6)	0.024 (4)	−0.003 (4)	−0.004 (3)	−0.010 (4)
C4	0.040 (5)	0.033 (4)	0.018 (3)	−0.004 (4)	0.000 (3)	−0.006 (4)
N11	0.036 (4)	0.053 (5)	0.026 (3)	−0.002 (4)	0.004 (3)	0.000 (3)
C12	0.030 (4)	0.050 (6)	0.024 (4)	0.004 (4)	−0.002 (3)	−0.003 (4)
C13	0.025 (4)	0.040 (5)	0.021 (4)	−0.002 (3)	0.003 (3)	−0.004 (3)
C14	0.032 (5)	0.039 (5)	0.022 (4)	−0.001 (4)	−0.002 (3)	0.001 (4)
C15	0.030 (5)	0.054 (5)	0.025 (4)	−0.001 (3)	−0.002 (4)	−0.002 (4)
C16	0.029 (4)	0.069 (7)	0.027 (4)	−0.001 (4)	0.006 (3)	−0.002 (4)
C21	0.025 (4)	0.044 (5)	0.019 (3)	0.002 (4)	0.001 (3)	0.000 (4)
C22	0.031 (5)	0.047 (6)	0.018 (4)	−0.001 (3)	0.001 (3)	0.002 (3)
N23	0.034 (4)	0.051 (4)	0.030 (3)	0.001 (3)	0.000 (3)	0.002 (3)
C24	0.030 (5)	0.065 (7)	0.029 (4)	−0.003 (4)	−0.005 (4)	−0.001 (4)
C25	0.028 (5)	0.063 (7)	0.030 (5)	0.004 (4)	−0.004 (4)	−0.006 (4)
C26	0.040 (4)	0.042 (5)	0.017 (4)	0.001 (4)	0.001 (3)	−0.002 (4)
B1	0.028 (4)	0.058 (6)	0.034 (5)	0.011 (5)	0.006 (4)	−0.003 (5)
F1	0.071 (5)	0.122 (7)	0.127 (7)	0.029 (5)	0.007 (5)	0.055 (5)
F2	0.057 (4)	0.103 (6)	0.136 (7)	0.016 (4)	0.042 (4)	0.000 (4)
F3	0.118 (6)	0.166 (7)	0.044 (4)	−0.038 (6)	0.003 (4)	−0.018 (5)
F4	0.079 (5)	0.118 (6)	0.095 (5)	−0.046 (4)	0.005 (4)	−0.025 (5)

### Geometric parameters (Å, º)

**Table:**

Ag1—N11	2.145 (6)	C13—C14	1.405 (11)
Ag1—N23^i^	2.155 (6)	C14—C15	1.382 (11)
Ag1—F1^ii^	3.168 (9)	C14—H14	0.9500
Ag1—F2	2.832 (8)	C15—C16	1.365 (11)
Ag1—F2^iii^	2.972 (7)	C15—H15	0.9500
O1—C1	1.210 (9)	C16—H16	0.9500
O2—C1	1.345 (9)	C21—C22	1.389 (10)
O2—C2	1.443 (9)	C21—C26	1.394 (11)
O3—C4	1.196 (9)	C22—N23	1.327 (10)
O4—C4	1.341 (9)	C22—H22	0.9500
O4—C3	1.458 (9)	N23—C24	1.352 (11)
C1—C13	1.504 (11)	N23—Ag1^iv^	2.155 (6)
C2—C3	1.492 (12)	C24—C25	1.382 (12)
C2—H2A	0.9900	C24—H24	0.9500
C2—H2B	0.9900	C25—C26	1.383 (12)
C3—H3A	0.9900	C25—H25	0.9500
C3—H3B	0.9900	C26—H26	0.9500
C4—C21	1.506 (10)	B1—F1	1.329 (13)
N11—C12	1.332 (10)	B1—F2	1.344 (11)
N11—C16	1.356 (11)	B1—F3	1.379 (12)
C12—C13	1.389 (11)	B1—F4	1.391 (13)
C12—H12	0.9500		
			
N11—Ag1—N23^i^	168.8 (3)	C13—C14—H14	121.5
C1—O2—C2	115.4 (6)	C16—C15—C14	120.1 (8)
C4—O4—C3	114.7 (6)	C16—C15—H15	119.9
O1—C1—O2	124.5 (7)	C14—C15—H15	119.9
O1—C1—C13	123.3 (7)	N11—C16—C15	122.6 (8)
O2—C1—C13	112.2 (7)	N11—C16—H16	118.7
O2—C2—C3	112.9 (7)	C15—C16—H16	118.7
O2—C2—H2A	109.0	C22—C21—C26	119.8 (7)
C3—C2—H2A	109.0	C22—C21—C4	117.0 (7)
O2—C2—H2B	109.0	C26—C21—C4	123.2 (6)
C3—C2—H2B	109.0	N23—C22—C21	121.9 (8)
H2A—C2—H2B	107.8	N23—C22—H22	119.0
O4—C3—C2	108.1 (7)	C21—C22—H22	119.0
O4—C3—H3A	110.1	C22—N23—C24	118.6 (7)
C2—C3—H3A	110.1	C22—N23—Ag1^iv^	123.5 (6)
O4—C3—H3B	110.1	C24—N23—Ag1^iv^	117.3 (5)
C2—C3—H3B	110.1	N23—C24—C25	122.7 (8)
H3A—C3—H3B	108.4	N23—C24—H24	118.7
O3—C4—O4	124.8 (7)	C25—C24—H24	118.7
O3—C4—C21	123.7 (7)	C24—C25—C26	119.1 (8)
O4—C4—C21	111.6 (6)	C24—C25—H25	120.4
C12—N11—C16	118.7 (7)	C26—C25—H25	120.4
C12—N11—Ag1	123.7 (5)	C25—C26—C21	117.9 (7)
C16—N11—Ag1	117.3 (5)	C25—C26—H26	121.1
N11—C12—C13	121.2 (8)	C21—C26—H26	121.1
N11—C12—H12	119.4	F1—B1—F2	111.1 (9)
C13—C12—H12	119.4	F1—B1—F3	108.1 (10)
C12—C13—C14	120.3 (7)	F2—B1—F3	108.0 (9)
C12—C13—C1	117.5 (7)	F1—B1—F4	110.8 (9)
C14—C13—C1	122.2 (7)	F2—B1—F4	113.1 (9)
C15—C14—C13	116.9 (7)	F3—B1—F4	105.3 (8)
C15—C14—H14	121.5		
			
C2—O2—C1—O1	2.8 (11)	C13—C14—C15—C16	−2.1 (13)
C2—O2—C1—C13	−176.4 (6)	C12—N11—C16—C15	1.8 (14)
C1—O2—C2—C3	−93.6 (8)	Ag1—N11—C16—C15	−172.7 (7)
C4—O4—C3—C2	−152.9 (7)	C14—C15—C16—N11	0.6 (14)
O2—C2—C3—O4	−63.9 (9)	O3—C4—C21—C22	8.2 (13)
C3—O4—C4—O3	3.4 (12)	O4—C4—C21—C22	−171.7 (7)
C3—O4—C4—C21	−176.7 (7)	O3—C4—C21—C26	−170.4 (9)
N23^i^—Ag1—N11—C12	−177.4 (12)	O4—C4—C21—C26	9.7 (12)
N23^i^—Ag1—N11—C16	−3.2 (18)	C26—C21—C22—N23	1.0 (13)
C16—N11—C12—C13	−2.5 (12)	C4—C21—C22—N23	−177.7 (8)
Ag1—N11—C12—C13	171.6 (6)	C21—C22—N23—C24	0.2 (12)
N11—C12—C13—C14	0.9 (13)	C21—C22—N23—Ag1^iv^	−170.8 (6)
N11—C12—C13—C1	179.9 (8)	C22—N23—C24—C25	−0.3 (13)
O1—C1—C13—C12	−14.4 (12)	Ag1^iv^—N23—C24—C25	171.3 (7)
O2—C1—C13—C12	164.9 (7)	N23—C24—C25—C26	−1.0 (14)
O1—C1—C13—C14	164.7 (8)	C24—C25—C26—C21	2.1 (14)
O2—C1—C13—C14	−16.1 (11)	C22—C21—C26—C25	−2.2 (13)
C12—C13—C14—C15	1.4 (12)	C4—C21—C26—C25	176.4 (8)
C1—C13—C14—C15	−177.6 (8)		

Symmetry codes: (i) *x*, −*y*+3/2, *z*−1/2; (ii) −*x*+1/2, *y*−1/2, *z*; (iii) −*x*+1/2, *y*+1/2, *z*; (iv) *x*, −*y*+3/2, *z*+1/2.

## References

[bb1] Blake, A. J., Champness, N. R., Hubberstey, P., Li, W. S., Withersby, M. A. & Schröder, M. (1999). *Coord. Chem. Rev.* **183**, 117–138.

[bb2] Blessing, R. H. (1995). *Acta Cryst.* A**51**, 33–38. 10.1107/s0108767394005726 7702794

[bb3] Brito, I., Vallejos, J., Cárdenas, A., López-Rodríguez, M., Bolte, M. & Llanos, J. (2011). *Inorg. Chem. Commun.* **14**, 897–901.

[bb4] Brito, I., Vallejos, J., López-Rodríguez, M. & Cárdenas, A. (2010). *Acta Cryst.* E**66**, o114. 10.1107/S1600536809052106 PMC2980043 21580003

[bb5] Sheldrick, G. M. (2008). *Acta Cryst.* A**64**, 112–122. 10.1107/S0108767307043930 18156677

[bb6] Spek, A. L. (2009). *Acta Cryst.* D**65**, 148–155. 10.1107/S090744490804362X PMC2631630 19171970

[bb7] Stoe & Cie (2001). *X-AREA* Stoe & Cie, Darmstadt, Germany.

